# Supplementary figures and images for: Postnatal plasticity in the olfactory system of the juvenile swine brain
Source: Brain Struct Funct. 2025 Oct 6;230(8):152. doi: 10.1007/s00429-025-03008-1 (PMC12500763; doi:10.1007/s00429-025-03008-1)

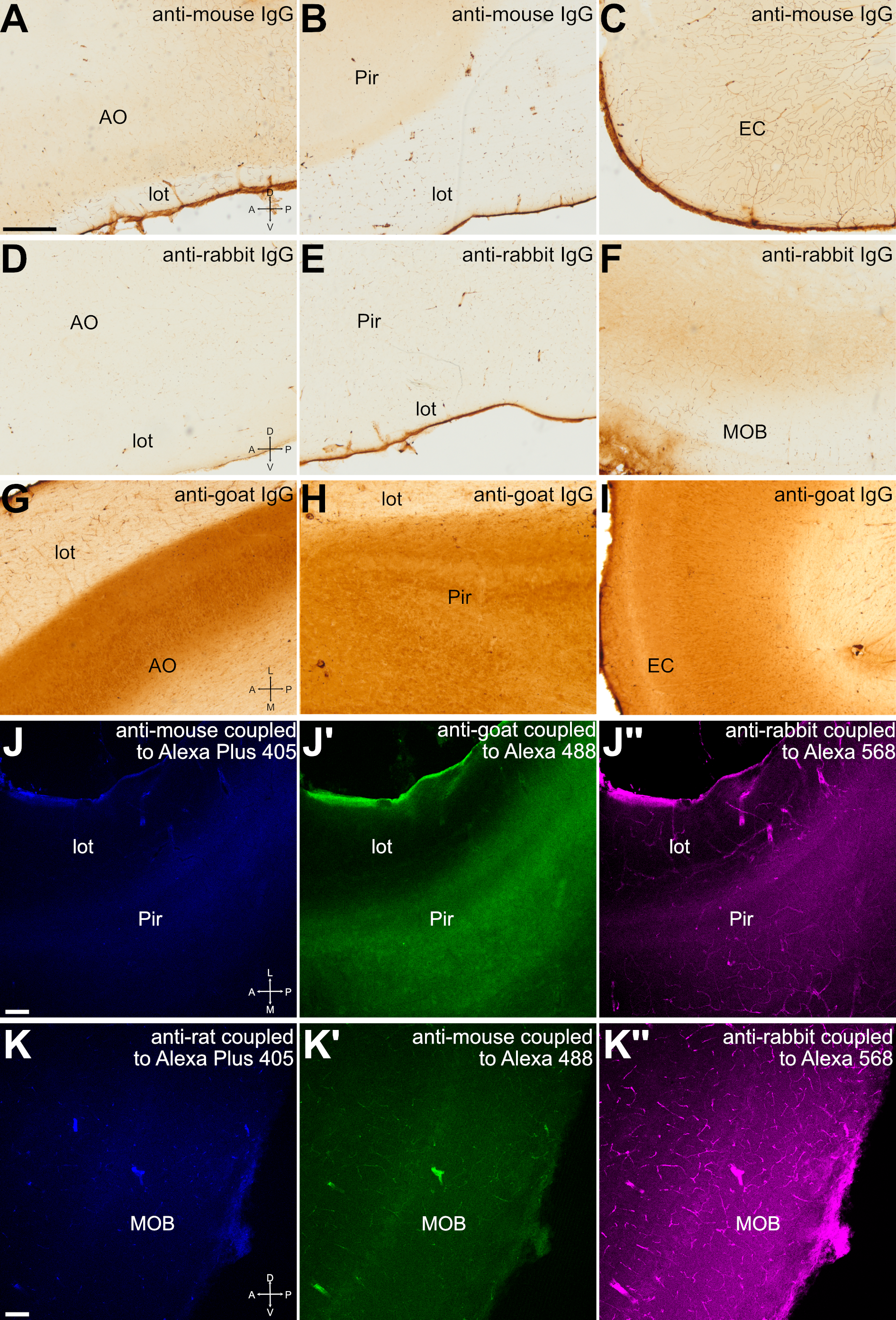

Supplement: Supplementary file 1 — Supplementary Material 1 Supplementary figure negative controls for immunohistochemistry and Immunofluorescence. Horizontal (A-C, G-J’’) and sagittal (D-F, K-K’’) sections at the level of anterior olfactory area (AO), piriform cortex (Pir), entorhinal cortex (EC), and main olfactory bulb (MOB), processed for immunohistochemistry (A-I) or triple immunofluorescence (J-K’’) omitting primary antibodies. No specific immunoreactivity was detected, although the anti-goat secondary antibodies produced some background (without clear cell labeling), possibly due to the phylogenetic proximity between goat and swine (G-I, J’). In addition, blood vessels were labeled with some of the secondary antibodies (C, G, I, J, J’’, K-K’’). Mediolateral and dorsoventral axes are indicated in A for orientation. For abbreviations, see list. Scales: A = 500 μm (applies to A-I); J = 100 μm (applies to J-K’’) [file 429_2025_3008_MOESM1_ESM.tiff]
